# Genome sequence of a lytic phage phi1_171026 targeting carbapenem-resistant *Enterobacter xiangfangensis*

**DOI:** 10.1128/mra.01335-25

**Published:** 2026-04-27

**Authors:** Yanling He, Yu Feng, Zhiyong Zong

**Affiliations:** 1 Center of Infectious Diseases, West China Hospital, Sichuan University 12530, Chengdu, China; 2 Division of Infectious Diseases, State Key Laboratory of Biotherapy, Chengdu, China; 3 Center for Pathogen Research, West China Hospital, Sichuan University 12530, Chengdu, China; Loyola University Chicago, Chicago, Illinois, USA

**Keywords:** *Enterobacter xiangfangensis*, carbapenem resistance, endolysin, phage, phage therapy

## Abstract

We describe a lytic phage phi1_171026 genome from *Certrevirus* genus within *Vequintavirinae* subfamily isolated from sewage and capable of lysing ST114-type carbapenem-resistant *Enterobacter xiangfangensis* strains. The genome is 151,090  bp in length, with a guanine-cytosine (GC) content of 50.90% and consisting of 324 protein-coding sequences, including two endolysin-encoding genes and 17 tRNAs.

## ANNOUNCEMENT

*Enterobacter* spp. is the third most common nosocomial pathogen in the family *Enterobacteriaceae* (1). Carbapenem-resistant *Enterobacter* strains are increasingly prevalent in clinical settings across China (2), especially *Enterobacter xiangfangensis* (3). Sequence type (ST) 114 is an epidemic clone of *E. xiangfangensis* carrying *bla*_NDM_ encoding New Delhi metallo-β-lactamase mediating broad-spectrum β-lactam resistance, including carbapenems (4, 5). Limited antimicrobial options for carbapenem-resistant *Enterobacter* infections have challenged clinical treatment (6). Bacteriophage (phage) therapy as a renewed anti-infection strategy offers a promising alternative for multidrug-resistant organisms. Endolysins, phage-encoded enzymes that hydrolyze host peptidoglycan, have emerged as potent antimicrobial agents (7). We present the genome of phage phi1_171026 carrying two distinct endolysin-encoding genes that effectively lyse ST114 clinical isolates.

In April 2023, phi1_171026 was isolated from untreated sewage of West China Hospital using a ST114-type carbapenem-resistant *E. xiangfangensis* clinical strain 040008 as a host. The sewage sample was filtered to remove debris and bacteria. The filtrate was mixed with 040008 in Luria-Bertani (LB) broth and incubated for 4 h at 37°C with shaking to propagate phages. Phage lysate was centrifuged, filtered, and then subjected to top-agar overlay with the host for plaque formation. Single plaques were purified through three rounds of isolation (8, 9). Phage morphology was observed using an HT7800 transmission electron microscope (Hitachi, Tokyo, Japan). Genomic DNA was extracted using the Phage DNA Isolation Kit (Norgen Biotek, Canada), and the sequencing library was prepared using the NEBNext Ultra II DNA Library Preparation Kit (New England Biolabs, MA), followed by sequencing on an Illumina NovaSeq 6000 platform (200× coverage). Genomic DNA was sheared to approximately 350 bp using a Covaris LE220R-plus ultrasonicator, with subsequent purification and size selection using Agencourt SPRIselect beads. Reads were trimmed for adapters using Trimmomatic v0.39 (10) and assembled with Unicycler v0.5.1 (11). Genome completeness and contamination were detected using CheckV v1.0.3 (12). The taxonomy of phi1_171026 was determined by BLASTn (https://blast.ncbi.nlm.nih.gov/Blast.cgi) analysis against the National Center for Biotechnology Information (NCBI) nt database and the closest match (highest identity × coverage) serving as the reference. The phage genome was annotated using Pharokka v1.8.1 (13). The lifestyle was predicted using PhaTYP v3.0 (14). Antimicrobial resistance and virulence genes were identified using the CARD v3.2.7 (15) and VFDB (18 August 2023) (16) databases. Default parameters were used unless specified.

**Fig 1:**
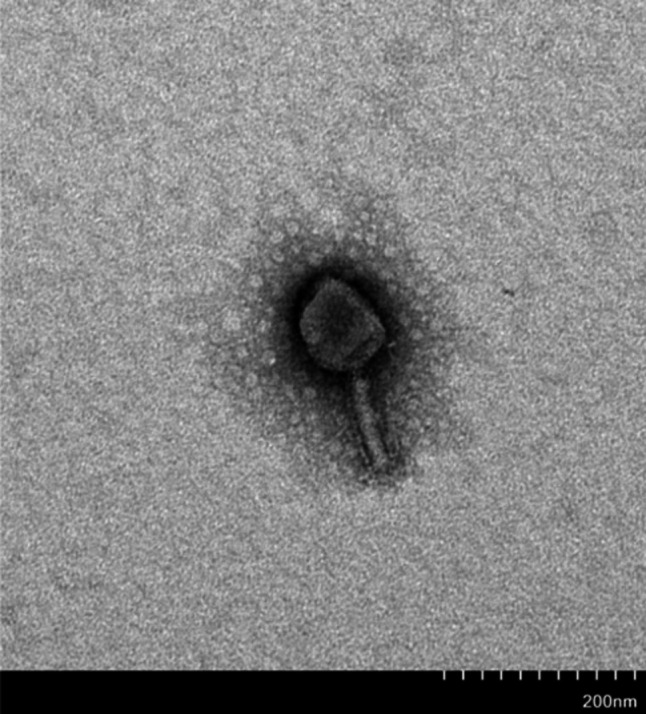
Micrographs of phage phi1_171026 under transmission electron microscopy. Scale bar represents 200 nm.

The phage possessed an icosahedral head (89 nm in diameter) and a contractile tail (105 nm in length) (Fig. 1). The 151,090-bp genome (50.90% GC) was generated from 7,418,962 paired-end 150-bp reads (1.11 Gb) after filtering raw reads through quality control. The result of BLASTn showed that phi1_171026 had the highest DNA similarity (88.43%, identity ×coverage) with *Enterobacter* phage vB_ExiM_F4M1E (accession no. OL744216.1), which belonged to the genus *Certrevirus* of the subfamily *Vequintavirinae*. The lytic lifestyle of phi1_171026 was predicted. A total of 324 coding sequences (CDSs) and 17 tRNAs were identified in this phage genome, including two conserved endolysin genes among this genus (17). No known antimicrobial resistance nor virulence gene was detected in its genome. These features of phi1_171026 indicate the potential suitability of this phage for biocontrol applications.

## Data Availability

The complete genome sequence of*Enterobacter xiangfangensis* phage phi1_171026 has been deposited in GenBank under accession number PX516826. The corresponding raw sequencing reads have been deposited in the Sequence Read Archive (SRA) under Run accession number SRR36157937. The version described in this paper is the first version.
